# Linkages between the Sustainable Development Goals and health in Somalia

**DOI:** 10.1186/s12889-024-18319-x

**Published:** 2024-03-27

**Authors:** Rage Adem, Hassan W. Nor, Mohamed M. Fuje, Abdinur H. Mohamed, Tobias Alfvén, Rhoda K. Wanyenze, Ahmed Y. Guled, Mohamed M. Biday, Nina Viberg, Daniel Helldén

**Affiliations:** 1 Benadir University Institute of Research & Development (BIRD), Mogadishu, Somalia; 2 Department of Global Public Health, Karolinska Institutet (KI), Stockholm, Sweden; 3 Center of Excellence for Sustainable Health (CESH), Karolinska Institutet (KI), Stockholm, Sweden; 4 Center of Excellence for Sustainable Health (CESH), Makerere University, Kampala, Uganda; 5 Department of Disease Control and Environmental Health, School of Public Health, Makerere University, Kampala, Uganda

**Keywords:** Sustainable development goals (SDGs), SDG linkages, Somalia, Health, Multi- sectoral collaboration

## Abstract

**Background:**

The Sustainable Development Goals (SDGs) adopted in 2015 compromises 17 universal and indivisible goals for sustainable development, however the interactions between the SDGs in Somalia is not known which is vital for understanding potential synergies and trad-offs between the SDGs. Hence, this study aims to identify and classify the linkages between the SDGs with a focus on health and well-being (SDG 3) in Somalia.

**Methods:**

Following the SDG Synergies approach, 35 leading experts from the public and private sectors as well as academia and civil society gathered for a 2-day workshop in Mogadishu and scored the interactions between the individual SDGs on a seven point-scale from − 3 to + 3. From this, a cross-impact matrix was created, and network models were used to showcase the direct and indirect interactions between the SDGs with a focus on SDG 3 (good health and well-being).

**Results:**

Many promoting and a few restricting interactions between the different SDGs were found. Overall, SDG 16 (peace, justice, and strong institutions) influenced the other SDGs the most. When second-order interactions were considered, progress on SDG 16 (peace, justice, and strong institutions) showed the largest positive impact on SDG 3 (good health and well-being). SDG 3 (good health and well-being) was heavily influenced by progress on other SDGs in Somalia and making progress on SDG 3 (good health and well-being) positively influenced progress on all other SDGs.

**Conclusion:**

The findings revealed that in Somalia, the interactions between the SDGs are mostly synergistic and that SDG 16 (peace, justice, and strong institutions) has a strong influence on progress on other SDGs as well as progress on SDG 3 (good health and well-being). This study highlights the need for a multisectoral strategy to accelerate progress on the SDGs in Somalia in general, and particularly SDG 3 (good health and well-being).

**Supplementary Information:**

The online version contains supplementary material available at 10.1186/s12889-024-18319-x.

## Background

The 2030 Agenda and the Sustainable Development Goals (SDGs) which was endorsed by all United Nations Member States in 2015 provides a shared roadmap for peace and prosperity for people and the planet now and in the future [1]. The 17 goals are an urgent call to action for all nations in a global partnership. The indivisibility and interconnectedness of the SDGs are stipulated in the 2030 Agenda, but there is little guidance on how the synergies and trade-offs between the goals or targets should be handled [2]. Although a developing field, the current research on the interactions between the SDGs is limited when it comes empirical grounding in a country setting [3]. Further, adopting and implementing the SDGs in countries that suffer from conflicts or institutional instability has proven hard, despite these challenges being at the heart of the 2030 Agenda in these settings [4]. With the adoption of the 2030 Agenda in 2015 and the first voluntary national report on the SDGs submitted to the United Nations in 2022, Somalia is accelerating the work to implement the 2030 Agenda and the SDGs [1]. The latest national development plan for Somalia showcase a strong emphasis on poverty reduction, inclusive and accountable politics, security and rule of law, economic growth and social development with the SDGs mapped and incorporated under these overarching pillars of priority [5].

In the most recent sustainable development report, Somalia ranked 160 out of 163 countries on the SDG Index [6]. The complexity, fragility, and general absence of SDG related statistics in the country make it hard to measure Somalia’s progress towards the attainment of the SDGs and specifically SDG 3 (good health and well-being). It stands clear however that Somalia will not be able to reach the health and nutrition related SDG targets by 2030 [7]. Descriptive information on available sustainable development some indicators are presented in Table 1. Somalia´s progress towards the 2030 Agenda is hindered by the 30-year state collapse, climate change, environmental degradation, a lack of investment in vital basic social and health care services and poor economic performance [8]. Over this time, various governance structures have failed to shield the population from conflicts, insecurity, and famines. The inability to cope with reoccurring shocks might be the major cause to why the trust in government, non-governmental organizations and international organizations is low in almost all provinces [9]. Additionally, there has been repeated breakdowns of infrastructure and lack of development on the broader social determinants of health which has stifled efforts of improving the health and well-being of the population. While recognizing the significant and complex challenges that exist, Somalia has made commendable progress over the last years in building and strengthening nascent state structures that endeavor to, in an inclusive and participatory manner, provide peace, security and justice; reduce corruption; and improve human rights [10]. Over the last years, the country´s economic growth has been on a positive trajectory despite the COVID-19 pandemic and droughts [11]. As made clear in the latest development plan, the Somali government focuses on developments and interventions that can make positive impacts across different sectors [5].

**Table 1:** Overview of selected sustainable development goal indicators over time in Somalia

**SDG**	**Indicator description**	**2000**	**2005**	**2010**	**2015**	**2019**	**Source**
**1** No poverty	Proportion of population living below the national poverty line (%)	43 (2002)				68 (2017)	Somalia Voluntary National Review Report 2022
**2** Zero hunger	Prevalence of undernourishment (% of population)	71 (2002)	71	70	60	53 (2020)	World Bank
**3** Good health and well-being	Under-five mortality rate, (deaths per 1,000 live births)	173	173	158	135	118	World Bank
	Proportion of children under five years moderately or severely stunted (%)			25 (2009)		28	Somalia Voluntary National Review Report 2022
	Proportion of births attended by skilled birth health personnel (%)	34	33 (2006)			32	Somalia Voluntary National Review Report 2022
**4** Quality education	Completion rate at primary level (%)		4 (2006)		16 (2016)		Somalia Voluntary National Review Report 2022
	Completion rate at secondary level (%)					65	Somalia Voluntary National Review Report 2022
**5** Gender equality	Proportion of seats held by women in legislation institutions (%)		8 (2006)	7 (2012)	14 (2015)	24 (2020)	Somalia Voluntary National Review Report 2022
**6** Clean water and sanitation	Proportion of population using basic drinking water services (%)		29 (2006)			66	Somalia Voluntary National Review Report 2022
	Proportion of population with basic sanitation services (%)					40	Somalia Voluntary National Review Report 2022
**7** Affordable and clean energy	Proportion of population with access to electricity	2	15	52	51	49	World Bank
**8** Decent work and economic growth	GDP per capita (current US$)	127 (1990)		350 (2013)	386	419	World Bank
**9** Industry, innovation and infrastructure	Proportion of population covered by at least 3G mobile network (%)			10 (2011)	39 (2016)	66 (2017)	Somalia Voluntary National Review Report 2022
**10** Reduced inequalities	Gini index disposable income (0-100)					37 (2017)	World Bank
**11** Sustainable cities and communities	Proportion of urban population living in slums (%)		74	74	74	72 (2018)	World Bank
**13** Climate action	Total greenhouse gas emission (kt of CO2 equivalent)	25	27	25	26 (2014)	25	World Bank
**14** Life below water	Average proportion of Marine Key Biodiversity Areas covered by protected areas	0%	0%	0%	0%	0%	World Bank
**15** Life on land	Forest area as a proportion of total land area (%)	12	11	11	10	10	World Bank
**16** Peace, justice and strong institutions	Voice and Accountability (ranges from approximately -2.5 (weak) to 2.5 (strong) governance performance)	-1,7	-1.8	-2	-2	-1.85	World Bank

The population of Somalia has endured protracted internal conflicts with devastating effects on the delivery of essential and lifesaving health care services. The health system in Somalia face the double burden of a flawed pre-conflict health system, characterized by deficiencies and inequities, and the long-term impact of conflict on the health status of the population and its resultant strain on the health system [12]. As a result of the prolonged fragility of Somalia, existing health infrastructures have been destroyed and effective institutional investment in quality health services has been prevented [13]. This extended humanitarian crisis in the country has heavily weakened the public health sector, causing high maternal and child mortality; heavy burden of communicable and non-communicable diseases, including mental disorders; and emergency levels of malnutrition [8]. The health sector in Somalia has been recovering slowly, and multiple challenges still exist [1]. The health system of Somalia is highly fragmented, and coverage of essential health services is very low, ranking second to last in the universal health coverage effective coverage index [14]. Life expectancy at birth in Somalia increased from 43 years in 1960 to 56 years in 2020 [15] while the under-five mortality rate was 115 per 1000 live births in 2020 [16] . The main health challenges that Somalia is struggling with are a continuously high burden from infectious diseases, undernutrition and maternal and child health related morbidity and mortality [10, 17].

Given the multitude of societal challenges in Somalia, progress on health in Somalia is dependent on progress in other sectors, and vice versa. In order to leverage the synergies between sectors and handle possible trade-offs in an effort to accelerate sustainable development in the country, it is crucial to understand how progress in different sectors or SDGs affect each other at a country level. This would also enable prioritization of efforts given limited resources. Therefore, we aimed to identify and classify the linkages between the SDGs with a focus on health and well-being (SDG 3) in Somalia.

## Methods

The SDG Synergies approach developed by the Stockholm Environment Institute was applied to identify, describe, and classify the linkages between the SDGs with a focus on health and well-being. It has been used in Cambodia to understand the linkages between the SDGs and child health [18], as well as in Sri Lanka and other middle-income settings to discuss priority setting of SDGs or SDG targets [19]. For more details on health-focused applications of the SDG Synergies approach see Helldén et al. [20] however the major advantages of the method is that is provides a middle ground between a pure quantitative or qualitative approach, and that it incorporate the subjectivity which is inherent in decision making processes and prioritisation. In short, the approach consists of three steps: (i) identification and selection of the relevant goals or targets (ii) assessment of the interactions between the selected goals or targets by a multistakeholder group and (iii) analysis of the direct and indirect effects through network theory.

### Identification and selection of the relevant goals or targets

The SDGs consist of 17 goals, but all goals have a set of targets linked to them. In total, there are 169 SDG targets, leading to almost 30 000 potentials pairwise interactions, hence it was not feasible to assess all potential interactions between the SDG targets in Somalia. After in-depth consultations in the research team, local partners and given the nature of the research aim concerned, it was deemed most relevant to keep the analysis at the goal level. The first 16 SDGs were selected. Importantly, SDG 17 (partnerships for the goals) was excluded as it was deemed too broad to assess in a relevant way. The 16 SDGs included in the analysis have 240 unique pairwise interactions among each other.

### Assessing the interactions

A two-day workshop on the 16th -17th of November 2021 was conducted in Mogadishu, Somalia to assess the interactions between the SDGs. In total, 35 participants from the public and private sector, as well as academia and civil society took part in the workshop (See Supplementary Material). The participants were purposively selected based on their knowledge of, and engagement in, the SDGs in Somalia at a country level, while also trying to ensure that expertise on all SDGs was covered.

Five groups of seven participants discussed direct interactions between the SDGs, guided by the question “In the Somalia context, if progress is made on SDG X, how does this influence progress on SDG Y?”. The groups arrived through a consensus approach at a score on a seven point-scale from − 3 (strongly restricting) to + 3 (strongly promoting). The groups provided a short motivation for each score. As a basis for the discussion the participants had their expert knowledge as well as fact sheets on each SDG based on the latest UN Somalia report [10]. Each group scored a set of interactions between specified SDGs and after all groups had scored their set of interactions the groups discussed and double-checked their own scoring. They further reviewed the scorings made by another group, and if they did not agree with the score given by the other group it was noted as a discrepancy. All discrepancies between the groups’ scorings were discussed in plenary setting, coming to a consensus score. When that was not possible, voting was used to arrive at a final score. The final scores and motivations were entered into tailor-made software [21] provided by Stockholm Environment Institute which included an intuitive data form as well the statistical methods necessary to conduct the analyses described below.

### Cross-impact matrix and network analysis

A cross-impact matrix was derived from the scores of the individual interactions. This provides an overview of the pattern of restricting, no influence and promoting interactions and showcase how each goal influence the other SDGs and vice versa. To gain a more systemic overview of the influence from individual interactions, it can be useful to move beyond direct interactions and also include indirect or second order interactions through network analysis. In this way, one can capture how progress (or lack thereof) can more holistically impact the network, in our case the SDGs in Somalia. A detailed expose of the mathematical properties are out of the scope of this article, we refer the interested reader to Newman [22] for a general introduction to network analysis and Weitz et al. [23, 24] for applicability within the SDG Synergies approach. In short, the net influence of a certain goal on the network as a whole including second order interactions can be described as:

$${I}_{g}^{Total}= {I}_{g}^{1st}+ \sum {I}^{2nd}= {D}_{g}^{Out}+ \sum _{j\ne g}{I}_{gj}{D}_{j}^{Out}$$

where ${\text{I}}_{\text{g}}^{1\text{s}\text{t}}$ is the influence of goal g on its closest neighbours, ${\text{I}}^{2\text{n}\text{d}}$ is the influence from g`s neighbour’s on their neighbours, ${\text{D}}_{\text{g}}^{\text{O}\text{u}\text{t}}$ is the out-degree (or influence) of goal g, ${\text{I}}_{\text{g}\text{j}}$ is the strength of interaction from goal g to goal j, and ${\text{D}}_{\text{j}}^{\text{O}\text{u}\text{t}}$ is the out-degree (or influence( of goal g. The second order influence of goal A on goal D can be calculated by ${I}_{A\to D}^{2nd}= \sum _{i}{w}_{Ai}{w}_{iD }$ in which *i* runs over all goals connecting *A* and *D*, and *w*_*i*_ is the strength of the interaction. A written informed consent form was signed by the workshop participants at the beginning, informing them that the results would be used for a publication. The study followed the ethical principles stipulated in the declaration of Helsinki and ethical approval from the Somalia National Institute of Health (NIH/IRB/05/OCT/2023), Benadir University Internal Ethical Review Board (BU/IERB/163/2021) as well as an advisory opinion from the Swedish Ethical Review Authority (Dnr 2021 − 0501).

## Results

### The interactions between the SDGs in Somalia

**Fig. 1:**
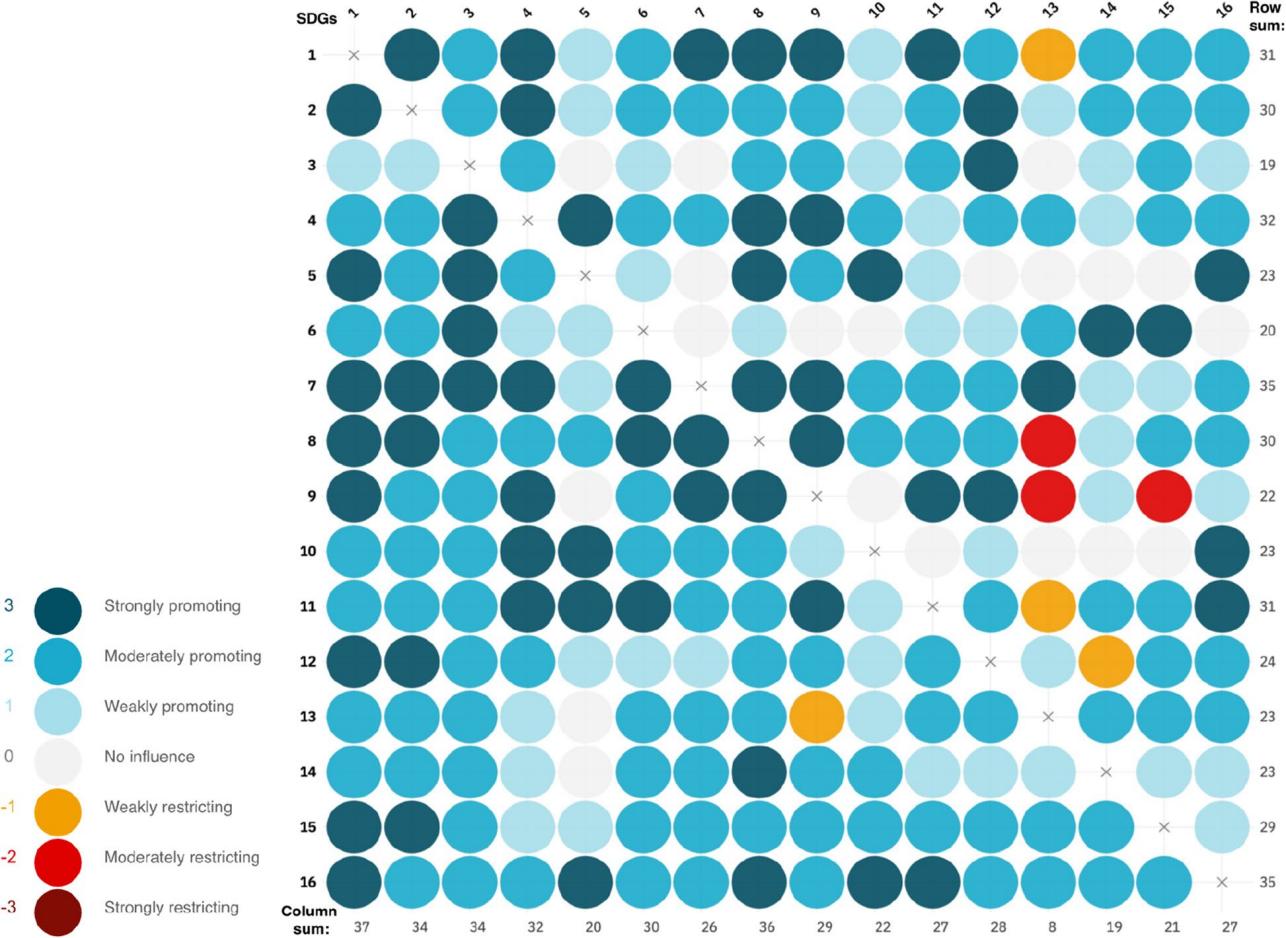
Cross-impact matrix of the 16 Sustainable Development Goals in Somalia. Dark blue color indicates a strongly promoting interaction, white indicate no influence and dark red a strongly restricting interaction. The rows in the figure show how the SDG influence other goals, while the column indicate how the SDG is influenced by other SDGs. For example in row 3 column 4, SDG 3 (good health and well-being) is moderately promoting SDG 4 (quality education). The row sum implies the net influence of the goal on the network, and the column sum show the how much the goal is influenced by all other goals in the network

The cross-impact matrix reveals many promoting and a few restricting interactions between the different SDGs (Fig. 1). Out of the 240 unique direct interactions, 7 were interpreted as restricting, 20 showed no influence and 213 were interpreted as promoting the progress of the other SDG. The row sum of the cross-impact matrix gives an estimate of the degree of influence one goal has on the network as a whole. In our study, SDG 16 (peace, justice and strong institutions) and SDG 7 (affordable and clean energy) have the largest overall effect on the network when considering the direct interactions. However, SDG 1 (no poverty) and SDG 8 (decent work and economic growth) were the goals which were the most influenced by the other SDGs as indicated by the column sums.

**Fig. 2:**
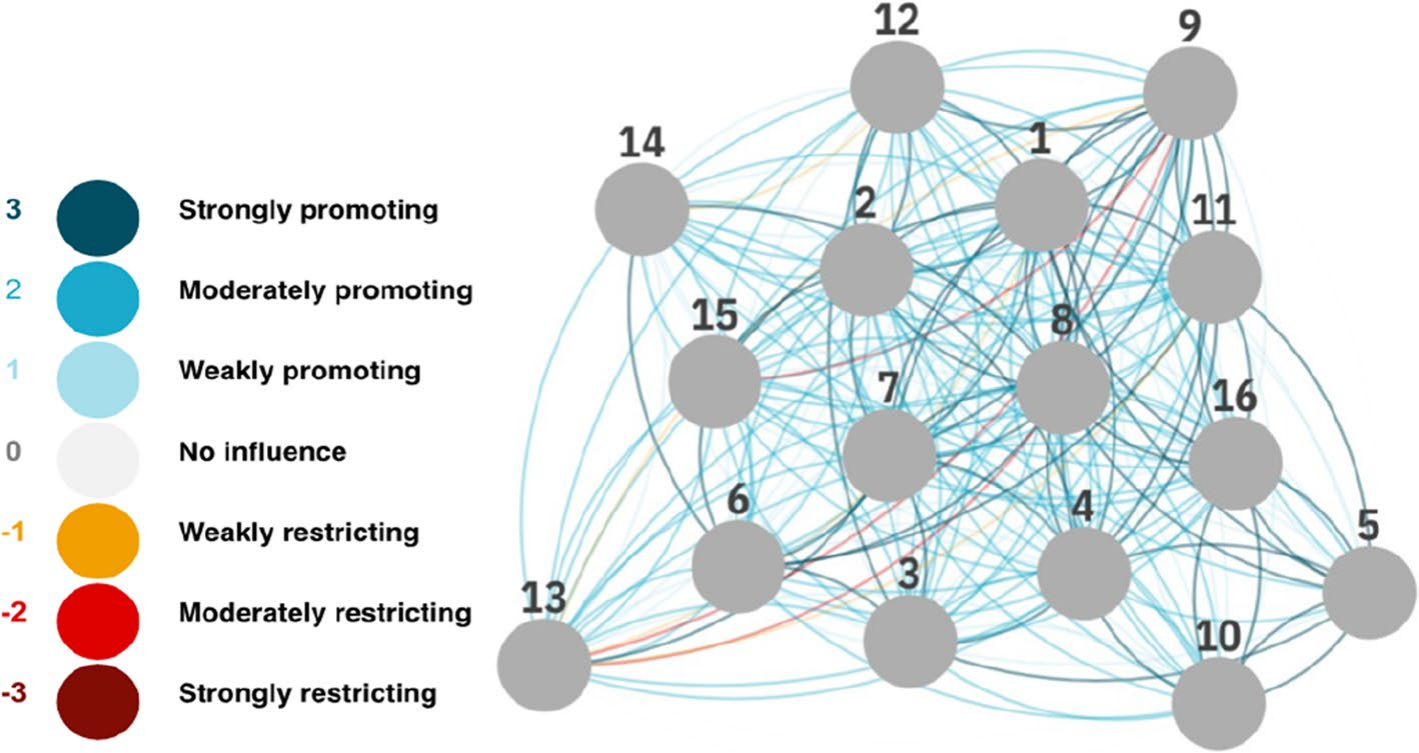
Network illustration of the direct interactions between the 16 sustainable development goals in Somalia

Most of the restricting interactions concerned SDG 13 (climate action), whereby participants considered that progress on SDG 1 (no poverty), SDG 8 (decent work and economic growth), SDG 9 (industry, innovation, and infrastructure) and SDG 11 (sustainable cities and communities) could lead to restricting the possibility to make progress on SDG 13 (climate action). The cross-impact matrix can also be illustrated as a network (Fig. 2) where SDG 13 (climate action) is more isolated due to the restrictive interactions.

By incorporating the second order interactions between the SDGs, we can understand how progress in one SDG can lead to indirect effects which provides a more relevant estimation of how an SDG influence the SDG network as a whole. Progress on one SDG can promote or restrict progress on another SDGs that in turn can promote or restrict influence other SDGs, when combining this first and second order interaction we can estimate the indirect effects. In Table 2, the ranking of the SDGs according to the row sum of the first order is provided as well as the ranking when considering second order row sum. When taking the second-order interactions into account, SDG 16 (peace, justice and strong institutions) becomes the single most influential SDG in the network although the SDGs move little in ranking.

**Table 2:** Direct and indirect interactions between the sustainable development goals in Somalia

Rank	SDG #	1^st^ First order row sum (Direct effects)	Rank	SDG #	2^nd^ Order row sum (Indirect effects)
1	SDG 7	35	1	SDG 16	963
1	SDG 16	35	2	SDG 7	957
2	SDG 4	32	3	SDG 1	894
3	SDG 1	31	4	SDG 11	869
3	SDG 11	31	5	SDG 4	868
4	SDG 2	30	6	SDG 8	853
4	SDG 8	30	7	SDG 2	848
5	SDG 15	29	8	SDG 15	802
6	SDG 12	24	9	SDG 12	704
7	SDG 5	23	10	SDG 13	670
7	SDG 10	23	11	SDG 10	669
7	SDG13	23	12	SDG 9	663
7	SDG 14	23	13	SDG 5	656
8	SDG 9	22	14	SDG 14	647
9	SDG 6	20	15	SDG 3	541
10	SDG 3	19	15	SDG 6	541

### Direct and indirect interactions between SDG 3 good health and well-being and the other SDGs

**Fig. 3:**
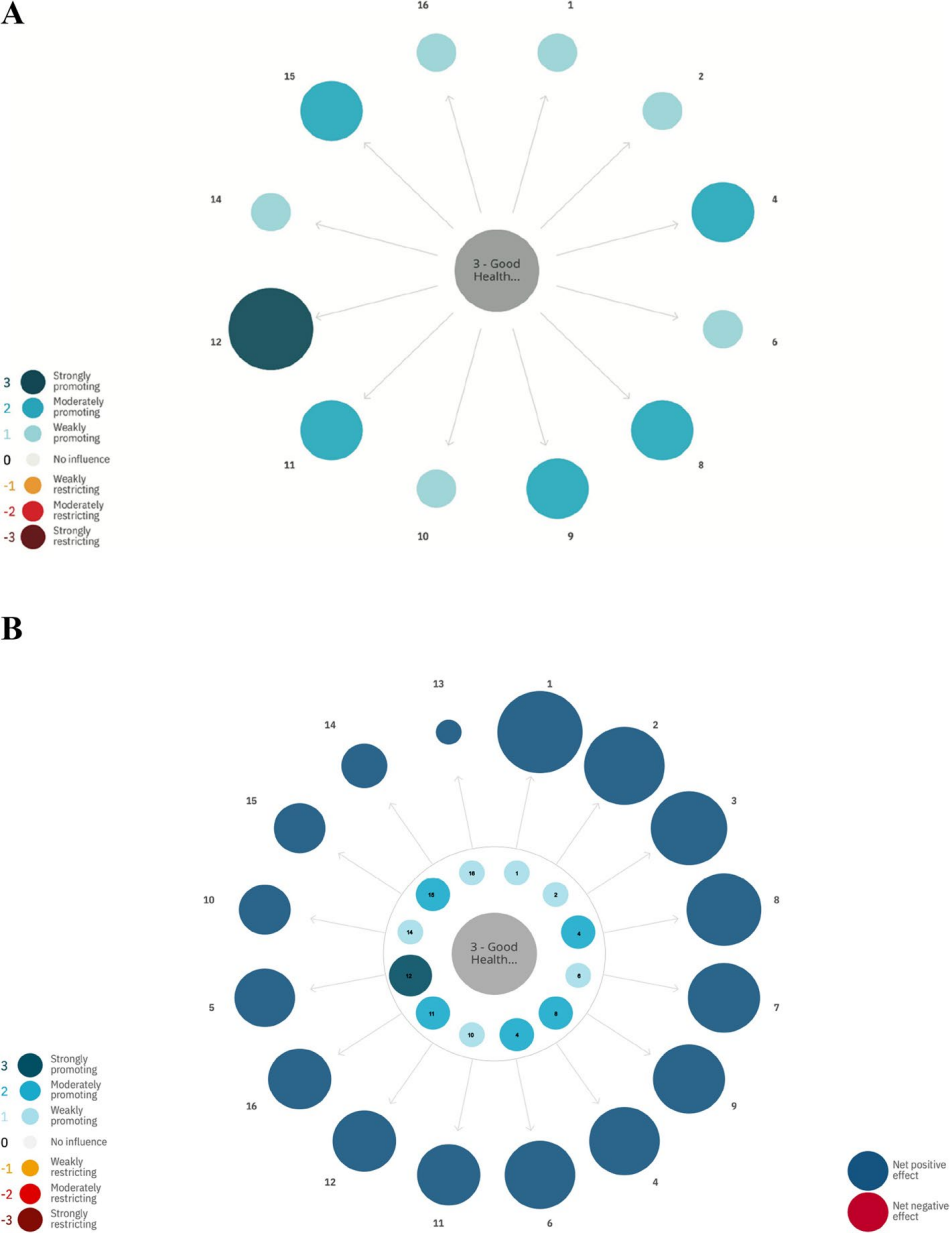
**A** Direct influence of progress on SDG 3 (good health and well-being) on other SDGs. The color and size of the bubbles is according to scale. Dark blue color indicates a strongly promoting interaction and dark red a strongly restricting interaction. **B** Indirect net influence of progress on SDG 3 (good health and well-being) on other SDGs. Color and size of bubbles according to scale. Dark blue color indicates a strongly promoting interaction and dark red a strongly restricting interaction

Regarding SDG 3 (good health and well-being), participants deemed that making progress on SDG 3 (good health and well-being) would lead to promoting the progress on virtually all other SDGs as illustrated in Fig. 3A. Further, considering the indirect interactions, progress on SDG 3 (good health and well-being) had the largest net positive influence on SDG 1 (no poverty) and SDG 2 (zero hunger). There also seems to be a positive feedback-loop, whereby progress on SDG 3 (good health and well-being) promote progress on other SDGs that in turn positively influence SDG 3 (good health and well-being). The smallest net positive influence was on environmental related SDGs (Fig. 3B).

**Fig. 4:**
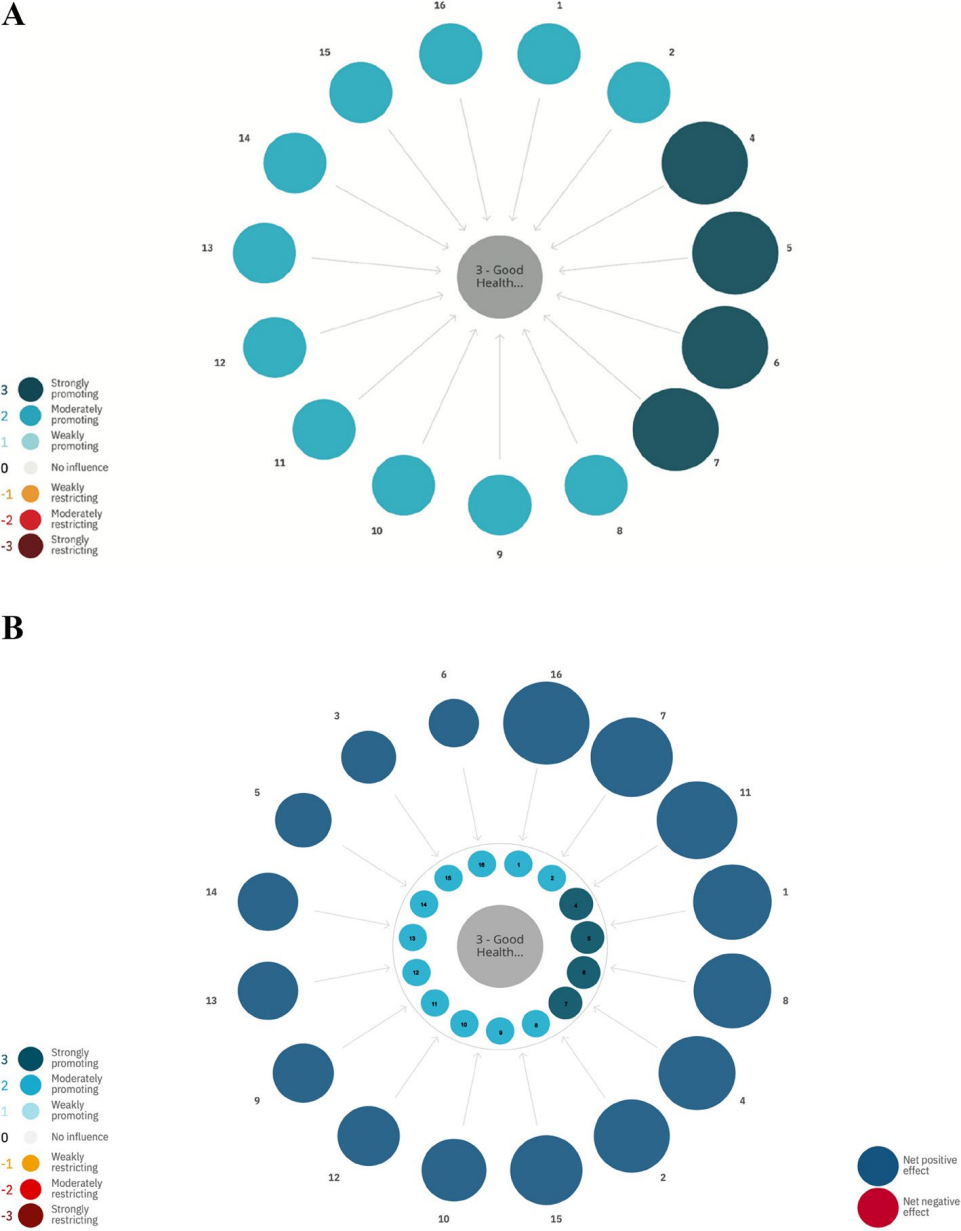
**A** Direct influence of progress on SDGs on SDG 3 (good health and well-being). The color and size of the bubbles is according to scale. Dark blue color indicates a strongly promoting interaction and dark red a strongly restricting interaction. **B** Indirect net influence of progress on SDG 3 from other SDGs. Color and size according to scale. Dark blue color indicates a strongly promoting interaction and dark red a strongly restricting interaction

When examining how other SDGs influence the possibility to make progress on SDG 3 (good health and well-being) it becomes evident that making progress on all other SDGs directly promote progress on SDG 3 (good health and well-being) (Fig. 4A). When considering indirect interactions SDG 16 (peace, justice and strong institutions) has the largest positive net influence on SDG 3 (good health and well-being) (Fig. 4B).

## Discussion

To our knowledge, this is the first study to systematically assess the linkages between SDGs in Somalia. The results indicate that of all the SDGs, making progress on SDG 16 (peace, justice, and strong institutions) would have the largest positive influence on work to achieve the 2030 Agenda in Somalia. SDG 3 (good health and well-being) was heavily positively influenced by progress on other SDGs. Further, making progress on SDG 3 (good health and well-being) has a positive feedback loop in which progress on SDG 3 (good health and well-being) enables improvements on other SDGs that in turn enable progress on SDG 3 (good health and well-being).

We found that SDG 16 (peace, justice and strong institutions) had the largest influence on the possibility to make progress on other SDGs and the 2030 Agenda. The critical role of institutional stability and rule of law for enabling sustainable development is well-known [25, 26], and while not a fast or easy process [27], it is key to unwinding the vicious cycle of conflict, poverty and instability in conflict affected countries [28]. A promising development is the re-engagement of World Bank in 2020 in Somalia and efforts to implement the first social security net system (Baxnaano) that aims to build resilience to external shocks and explicitly build institutional capacity within the government sector. Baxnaano’s primary goal is to shift from prolonged humanitarian assistance to a government-led, shock-responsive safety net system. This transition aims to combat chronic poverty, foster human development, and bolster households’ resilience to shocks. The strategy involves international collaboration to reinforce government systems, transforming current cash transfers into a sustainable social safety net aligned with the country partnership framework for scalability and reliability [29]. The effort to provide a social security net system, building trust and stronger institutions, is in line with the findings of our study that it would be beneficial to promote progress on SDG 16 (peace, justice and strong institutions) as this leads to the most influential promoting interactions for other SDGs and general sustainable development. Additionally, SDG 7 (affordable and clean energy) was shown to be promoting progress on all other SDGs and being the second most influential taking into considerations indirect interactions. This might reflect the current lack of access to energy, with only about half of the population having access to electricity and the access being further limited by frequent outages. This hinders all aspects of sustainable development, in particular as it almost exclusively relies on fossil fuel for its generation [30]. It is noteworthy that, making progress on SDG 13 (climate action) was deemed to be restricted by progress on SDG 1 (no poverty), SDG 8 (decent work and economic growth), SDG 9 (industry, innovation and infrastructure) and SDG 11 (sustainable cities and communities). Climate change over the last three decades has led to an increased strain on crop production in Somalia [31], while the increase in frequency of extreme weather events such as droughts exacerbates long-term climate change projections of increasing temperatures and lower precipitation [32]. Given the fundamental impact of climate change in Somalia on sustainable development, it is critical that the identified trade-off between climate change, poverty reduction, economic growth and infrastructure development must be carefully considered to ensure that the possible negative impacts of climate change mitigation and adaptation are minimized [33].

Our findings demonstrate that there are direct and indirect interactions between the SDGs in Somalia, this interconnectedness leads to the necessity of collaboration across sectors and should be considered in order to understand how to most effectively accelerate progress towards the 2030 Agenda in Somalia. The absence of government throughout the civil war and the subsequent years of widespread conflict resulted in the deterioration of Somalia’s public sector health system to virtual non-existence, making collaboration across sectors even more essential [34]. Despite the multitude of challenges, Somalia has made progress on improving the health and well-being in the country [35]. Similarly to other analyses of the linkages between health and the SDGs [20, 36, 37], we found that improving good health and well-being can enable progress on other SDGs. Conversely, health was deemed to be relying on the development of other sectors possibly due to the chronic instability of Somalia and relatively underdeveloped other sectors. Indeed, the major limitations identified to further improvement of health service delivery to women and children over the last two decades include persistent commodity and human resources shortages, poor infrastructure and limited access to vulnerable populations due to instability and conflict [34]. The fact that SDG 3 (good health and well-being) was deemed the least influential SDG taking into account direct and indirect interactions could be interpreted that albeit good health and well-being is essential for sustainable development, in a setting such as Somalia the impact of making progress on SDG 3 (good health and well-being) on the network as a whole, reflecting the broader SDG goals, is largely dependent of the state of SDG 3 (good health and well-being). If a population has relatively worse health outcomes and low well-being, it is likely that making progress on SDG 3 (good health and well-being) alone has a limited possibility to singlehandedly promote broader sustainable development but must be combined with other efforts. This emphasizes the need for a comprehensive understanding of the interdependencies between goals and underscores the importance of a synergistic approach to sustainable development that takes into account the broader social, economic, and environmental determinants of health. Concrete multisectoral collaborations between government, non-governmental and private sector actors will be necessary to overcome these barriers and provide a foundation for lasting systematic change [38]. An example of successful multisectoral collaboration is the Scaling Up Nutrition Movement, which has led to an increased effort to combat malnutrition through a multisectoral platform of the Somalia Multisectoral Nutrition Strategy, the strategy offers guidelines for the government, stakeholders, and various organizations. It emphasizes cross-sectoral integration and outlines several concrete objectives to improve the nutrition status of pregnant women, mothers, and children under five through actions in the health sector and collaboration with other sectors [39]. Perhaps unsurprisingly, SDG 16 (peace, justice and strong institutions) was deemed the most important SDG for promoting progress on SDG 3 (good health and well-being) in Somalia. The complex and evolving conflict setting in the country has been shown to directly and indirectly affect various health areas such as child undernutrition [40], mental health [41] and the COVID-19 pandemic [42]. Similarly devastating is the prevalent corruption in the health system [43] which leads to the breakdown of trust and worsening of health inequities [44] with people preferring private health care systems [45]. With peace, justice and strong institutions being at the core of a functioning health system [10], the importance of SDG 16 (peace, justice and strong institutions) for allowing the fragmented health system to heal cannot be overstated. Key strategies identified for improving the health system include ensuring health service for all, a focus on community participation and ownership as well as utilizing academic networks to promote and sustain educational and research capacity [8]. Further, developing a functioning workforce, health information and financing systems coupled with strengthening of leadership and governance are essential health system components that could accelerate improvements in SDG 3 (good health and well-being) in Somalia [12].

The main limitation of this study is that since there exist very limited quantitative data on developmental indicators from Somalia, it is hard to complement the more qualitative SDG Synergies approach used in this study with quantitative analysis triangulation. On the other hand, since the scoring is based on the country specific experience from participants working with these critical issues in Somalia the findings provide novel insights not possible to capture in pure qualitative or quantitative fashion. The SDG Synergies approach explicitly acknowledges that decision making include many different biases and is often highly subjective [19, 46, 47], and incorporating this subjectivity is often essential for understanding complex systems [47, 48]. The participatory approach of the actual scoring itself led to constructive discussions between experts from sectors that do not usually meet each other and helped to frame issues in a common language emphasizing the importance of a continuous dialogue to create mutual understanding between different sectors in order for multisectoral collaborations to succeed. Additionally, one limitation was that the analysis was focused on Somalia at a country level which might lead the findings to not be directly transferable to sub-country regions. Further the scoring of interactions was done at the goal level of the SDGs. Although this allowed for a more generalizable approach to SDG interactions in Somalia and help to build on the emerging evidence of interactions between the SDGs, we recommend further research focusing on a specific sub-region and a carefully selected set of SDG indicators to yield further actionable insights.

## Conclusions

The findings illustrate the importance of a multisectoral approach to accelerate work towards achieving the SDGs in Somalia. Concretely, we conclude that to make progress on SDG 3 (good health and well-being), it is important to consider interactions between SDG 3 (good health and well-being) and other SDGs to leverage synergies and carefully handle trade-offs when implementing concrete action. Given that progress on SDG 3 (good health and well-being) is heavily positively influenced by progress on other SDGs it becomes imperative to collaborate between many different sectors. Notably, intensified progress on SDG 16 (peace, justice and strong institutions) has the largest potential to positively impact the achievement of the 2030 Agenda in Somalia, both overall and for and SDG 3 (good health and well-being) specifically.

### Supplementary Information

**Supplementary Material 1.**

## Data Availability

All data generated or analysed during this study are included in this published article.

## Abbreviation

SDGs: Sustainable Development Goals
