# Supplementary material for: Linkages between the Sustainable Development Goals and health in Somalia
Source: BMC Public Health. 2024 Mar 27;24:904. doi: 10.1186/s12889-024-18319-x (PMC10967222; doi:10.1186/s12889-024-18319-x)
Supplement: Supplementary file 1 — Supplementary Material 1. [file 12889_2024_18319_MOESM1_ESM.docx]

**Supplementary Material**

We are in this together - linkages between Sustainable Development Goals and health in Somalia

**1. Stakeholders and paricipants in workshop**

**Note on participant sampling**

The research team identified a large number of individuals who according to the research team had knowledge of and engaged with SDG implementation in Somalia at a the country level. To ensure that as many different stakeholders as possible would be present (including a relatively balanced group with participants from government, non-governmental and civil society stakeholders) a selected number of individuals were invited to the workshop. The total number of participants were set at a maximum of 35 as it would otherwise be too difficult to have in-depth group and plenary discussion on individual interactions. Additionally, care was taken so that knowledge of and engagement with all SDGs at the country level was covered by the invited participants.

**Table S1.** **List of stakeholders participated in the workshop**

| Organization | Type of Organization | Number of Participants (N=35) |
| --- | --- | --- |
| Ministry of Planning & Economic Development | Governmental | 1 |
| Ministry of Agriculture & Irrigation | Governmental | 2 |
| Ministry of Health - Federal | Governmental | 2 |
| Ministry of Humanitarian & Disaster Management | Governmental | 1 |
| Ministry of Foreign Affairs | Governmental | 1 |
| Ministry of Health– Galmudug State | Governmental | 2 |
| Ministry of Water & Energy Resources | Governmental | 2 |
| Somali National University | Governmental | 2 |
| IOM – Somalia Office | Non – Governmental | 1 |
| UNICEF – Somalia Office | Non – Governmental | 1 |
| SOS – CV Somalia | Non – Governmental | 2 |
| Save the Children – Somalia Office | Non – Governmental | 1 |
| IIDA Women Development | Civil Society | 1 |
| SIMAD University | Academia | 2 |
| Mogadishu University | Academia | 2 |
| iRise – Hub | Non – Governmental | 1 |
| Somali Gender Hub | Civil Society | 1 |
| Horn Center | Civil Society | 1 |
| Tawakal Youth Group | Civil Society | 1 |
| Benadir University Faculty of Health Science | Academia | 2 |
| Benadir University of Medicine | Academia | 2 |
| BU-MISH Alumni | Academia | 1 |
| Somali Disaster Resilience Institute | Civil Society | 2 |
| Somali National Statistics Bureau | Governmental | 1 |

**2. SDG Synergies data collection form**

**
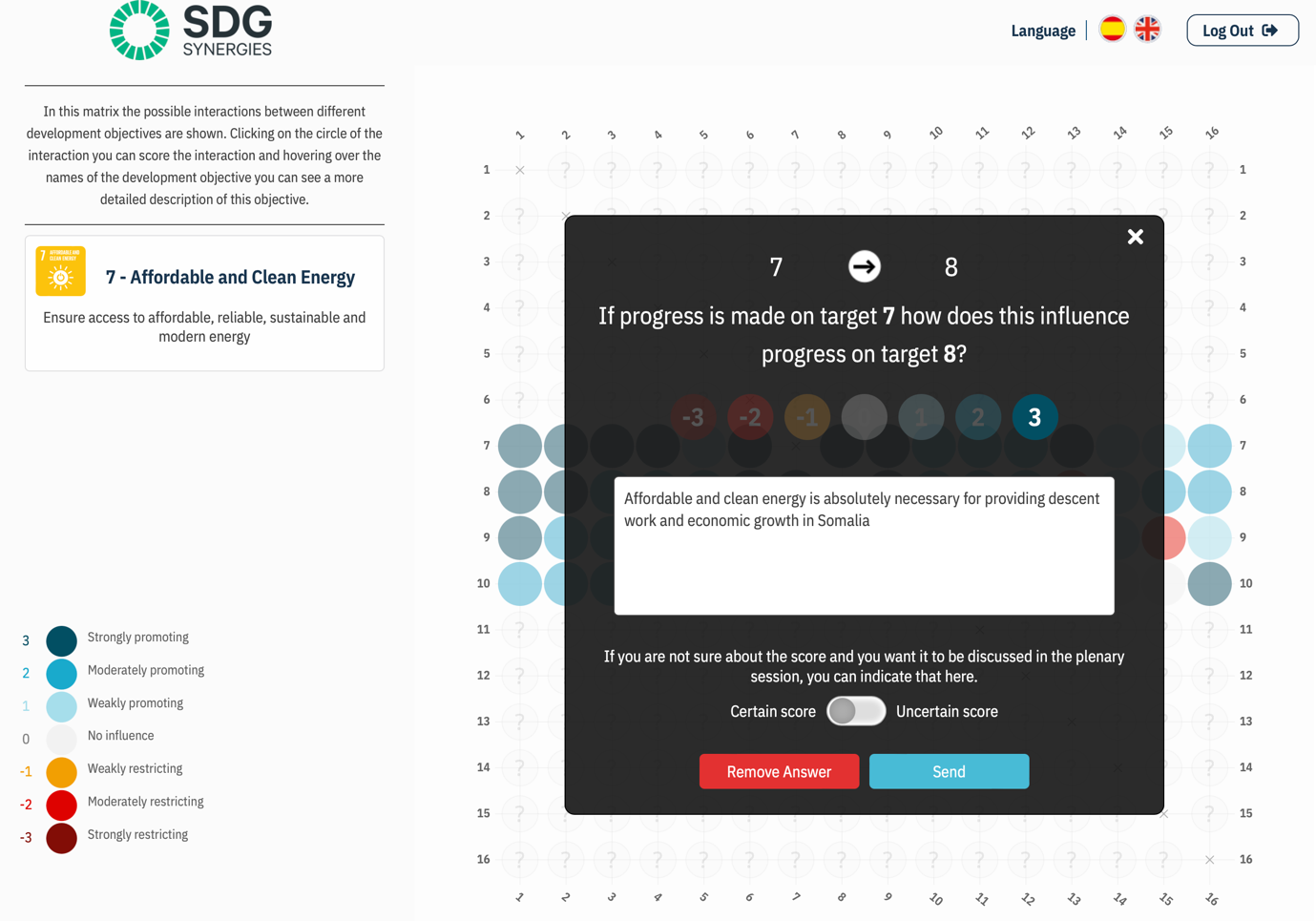
**

**Figure S1. Example data entry from in SDG Synergies website**
